# Exploring the Complexity of Processing-Induced Dehydration during Hot Melt Extrusion Using In-Line Raman Spectroscopy

**DOI:** 10.3390/pharmaceutics12020116

**Published:** 2020-02-01

**Authors:** Lærke Arnfast, Jeroen van Renterghem, Johanna Aho, Johan Bøtker, Dhara Raijada, Stefania Baldursdóttir, Thomas De Beer, Jukka Rantanen

**Affiliations:** 1 Department of Pharmacy, Faculty of Health and Medical Sciences, University of Copenhagen, Universitetsparken 2, 2100 Copenhagen, Denmark; l.arnfast@gmail.com (L.A.); dingeansich@gmail.com (J.A.); johan.botker@sund.ku.dk (J.B.); dhara.niper@gmail.com (D.R.); dr.stefania@gmail.com (S.B.); 2 Department of Pharmaceutical Analysis, Faculty of Pharmaceutical Sciences, Ghent University, Ottergemsesteenweg 460, B-9000 Ghent, Belgium; jeroenvanrenterghem@hotmail.be (J.v.R.); Thomas.DeBeer@UGent.be (T.D.B.)

**Keywords:** dehydration, hydrates/solvates, kinetics, Raman spectroscopy, extrusion, processing, preformulation, solid state stability, transformation

## Abstract

The specific aim in this study was to understand the effect of critical process parameters on the solid form composition of model drug compounds during hot melt extrusion using in-line Raman spectroscopy combined with Multivariate Curve Resolution-Alternating Least Squares (MCR-ALS) modeling for semi-quantitative kinetic profiling. It was observed that the hydrate and anhydrate solid forms of two model drugs in the melts of nitrofurantoin (NF):polyethylene oxide (PEO) and piroxicam (PRX):PEO could be resolved from a MCR-ALS model without an external calibration dataset. Based on this model, the influence of two critical process parameters (shear and temperature) on the solid form composition could be evaluated in a real-time mode and the kinetics of complex transformation pathways could be explored. Additionally, the dehydration pathways of NF monohydrate and PRX monohydrate in molten PEO could be derived. It can be concluded that dehydration of both hydrates in PEO occurs via competing mechanisms—a solution-mediated transformation pathway and a solid–solid transformation, and that the balance between these mechanisms is determined by the combined effect of both temperature and shear. Another important observation was that the water released from these hydrate compounds has a detectable effect on the rheological characteristics of this mixture.

## 1. Introduction

Hot melt extrusion (HME) is a well-established approach for manufacturing of innovative oral solid pharmaceutical dosage forms [1]. This approach is an inherently continuous and scalable process [2], which makes it an attractive alternative for new drug products. Additionally, HME is an upstream process for innovative products based on additive manufacturing principles and there is a wide range of futuristic ideas pointing towards the development of totally new types of pharmaceutical products [3,4,5]. There are several new marketed products that are based on HME and especially some of the new antiviral products based on this technology indicate that this method is an attractive approach for products containing several active compounds. These systems with multiple compounds can potentially be formulated by utilizing the whole solid form diversity, and HME has been reported to be a scalable production method for diverse multicomponent systems including both co-crystalline [6] as well as co-amorphous products [7,8]. This development is underpinning the importance of robust process understanding, and, where found necessary, robust process monitoring and control solutions. Molten polymeric mixture containing dissolved drug interacting with both polymer(s) and co-former(s), as well as solid-single component and multicomponent-drug particles is an extra-ordinary challenging system. Thermodynamic landscape of these systems should be carefully explored using diverse solid-state analytical methods building up towards constructing a hot melt extrusion process operating design space [9].

Critical process parameters in melt extrusion that may influence the solid form composition of the drug product include, most importantly, thermal and mechanical stress and these can also have multiplicative effects, such as viscous heating, experienced in the processing of high-viscous melts [10]. Real-time monitoring of these phenomena is important and both near infrared (NIR) and Raman spectroscopy have been investigated as process analytical technologies (PAT) for in-line drug concentration and solid-state monitoring during melt extrusion [11]. Multivariate regression models play an important role in the analysis of spectroscopic data for qualitative or semi-quantitative interpretation [12,13]. Collecting in-line Raman spectra from different barrel sections has been shown to give valuable information on the interaction between screw speed, temperature profile, and resulting solid form composition of the drug in the melt [14]. By combining a realistic and scalable process environment [15] with the opportunity to continuously collect process data in a highly adjustable time interval, mini-extruders with recirculation channels provide an opportunity for gaining mechanistic understanding of solid form transformations induced during melt extrusion. Interaction between drug and polymeric excipient(s) may cause solid form changes [16,17]. It may be hypothesized that different solid forms may display different degrees of solubility, as well as miscibility, with the given polymer. This is even more complicated when considering the effect of processing-induced stress, such as temperature and mechanical stress during HME. Indeed, we have observed that while nitrofurantoin anhydrate (NFAH) (form β) is practically insoluble in polyethylene oxide (PEO) at 70 °C, the nitrofurantoin monohydrate form (NFMH) dehydrates at this temperature when in contact with this molten polymer, a process temperature 50 °C below the expected off-line dehydration temperature (120 °C). In a previous study, we observed and suggested mechanism for this processing-induced dehydration [18].

Combining in-line Raman spectroscopy and Principal Component Analysis (PCA), it is possible to determine the effects of process parameters on the crystalline-to-amorphous transformation rate of a drug in a melt extrusion process, as shown in a recent study, where the amorphization of metoprolol in Eudragit^®^ RSPO was monitored as a function of screw speed [19]. Recently, a combination of Raman spectroscopy and Multivariate Curve Resolution (MCR) was found to be able to estimate the percentage of crystalline and molten polymer in a semi-crystalline polymer sample [20]. Furthermore, the MCR decomposition method requires no external calibration data, and, is a well-known data analytical technique for deconvolution of on-line and off-line monitoring of chemical reactions [21,22]. MCR offers an estimation of pure analyte signals and can thus provide quantification of the individual components in complex mixtures and their individual compound spectra, even in situations with limited prior information [21,23]. MCR has previously been applied to variable temperature diffraction data for exploration of the solid forms appearing during the dehydration of an atorvastatin calcium salt hydrate, and the kinetics of this transformation [24], where it was found that MCR provided a reliable tool for quick qualitative and quantitative assessment of phase transformations studied by X-ray powder diffraction.

Building on this, the aim of this study is to provide an insight into solid form diversity during HME processing, and specifically to utilize in-line Raman spectroscopy as a tool to explore the solid forms present during melt processing. Additionally, the influence of two critical process parameters, screw speed and temperature, on the solid form landscape is studied with a semi-quantitative kinetic profiling approach.

## 2. Experimental

### 2.1. Materials

Polyethylene oxide PEO, analytical grade, Mw: 100.000 g/mol, T_g_ −67 °C, T_m_ 65 °C) was acquired from Sigma-Aldrich (Saint Louis, MO, USA) and used as received. Nitrofurantoin monohydrate (NFMH) was recrystallized by dissolving nitrofurantoin anhydrate (NFAH, Ph. Eur., T_g_ 106 °C, T_m_ 270 °C) (acquired from UNIKEM, Copenhagen, Denmark) in water at ambient temperature, stirring the solution for 24 h, followed by filtration and drying of the slurry at ambient temperature and pressure. Full transformation to the monohydrate form was confirmed by powder X-ray diffraction (XRPD) analysis (CSD refcode: HAXBUD) (Figure S1). NFAH with a comparable particle size distribution was generated by storing NFMH in an oven at 130 °C and ambient pressure overnight. Full conversion to the anhydrate was confirmed from XRPD analysis (CSD refcode: LABJON01) (Figure S1). Piroxicam monohydrate (PRXMH) was recrystallized by dissolving piroxicam anhydrate (PRXAH, Ph. Eur., T_g_ 63 °C, T_m_ 201 °C) (acquired from Chr. Olesen Pharmaceuticals A/S, Gentofte, Denmark) in water at ambient temperature, stirring the solution for 24 h, followed by filtration and drying of the slurry at ambient temperature and pressure. Full transformation to the monohydrate form was confirmed from XRPD analysis (CSD refcode: CIDYAP) (Figure S1). PRXAH with a comparable particle size distribution was generated from drying PRXMH in an oven at 130 °C and ambient pressure overnight. Full conversion to the anhydrate was confirmed from XRPD analysis (CSD refcode: BIYSEH01) (Figure S1). Raman spectra of the materials were recorded at ambient temperature using a Kaiser Raman RxN2 spectrometer (Kaiser optical systems, Ann Arbor, MI, USA) equipped with a CCD detector.

### 2.2. Methods

#### 2.2.1. Extrusion

Sample of 6 g of physical mixtures of 15 wt% drug and 85 wt% PEO were extruded on a P0harma Melt Extruder (PME5, Xplore Instruments, Geleen, The Netherlands) with a total barrel volume of 5 mL, equipped with conical, fully intermeshing co-rotating twin screws of 120 mm length and 5.3–14 mm in diameter. This lab-scale melt extruder can be operated either in a continuous extrusion mode or in recirculation mode by switching of a valve at the die; in this study all extrusions were performed in recirculation mode. The recirculation time for all experiments was 10 min with a spectrum collected every 5 s. Two temperatures, 70 and 90 °C, and two screw speeds, 25 and 50 rpm, were employed. Each formulation was extruded in triplicate at each combination of temperature and screw speed. The logged data (i.e., barrel temperature, screw speed and the force exerted on the screws) from the mini extruder was captured by the Micro Compounder v10.1 software (Xplore Instruments, Geleen, The Netherlands).

#### 2.2.2. In-Line Raman Spectroscopy, Data Acquisition and Processing

A Raman Dynisco probe (Kaiser optical systems, Ann Arbor, MI, USA) was installed facing the screws at the bottom of the barrel (position 2) in a splittable barrel, using the setup previously reported [19]. The probe was connected via a fiber optical cable to the RxN2 Raman spectrometer (Kaiser optical systems, Ann Arbor, MI, USA), equipped with a CCD detector. In-line Raman spectra were collected every 5 s with an acquisition time of 3 s for duration of 10 min. The applied wavelength was 785 nm. Data from each experiment were assembled in two row-wise augmented matrices, one for PRX-PEO experiments and one for NF-PEO experiments. Variables from 1650 cm^−1^ to 930 cm^−1^ were included in the NF-PEO dataset, variables from 1710 cm^−1^ to 950 cm^−1^ were included in the PRX-PEO dataset. These spectral areas were selected to exclude regions of high noise and low signal. The first 4–9 samples of each run (from the beginning of the extrusion process) were discarded due to inadequate signal. Two experiments—NFMH 50 rpm 70 °C run 1 and PRXAH 50 rpm 70 °C run 2—were excluded from the model development because of a large number of noisy or low-intensity spectra in these datasets. Baseline correction (weighted least squares algorithm with a second order polynomial) was performed to minimize artifacts due to fluorescence and differences in offset. No further pre-processing was used.

The concentration profiles of the NF/PRX compounds in the melts during the extrusion experiments were resolved from their respective augmented matrices using the Multivariate Curve Resolution-Alternating Least Squares (MCR-ALS) algorithm from PLS_Toolbox 7.5 (Eigenvector Research Inc., Wenatchee, WA, USA), for Matlab. Constraints of non-negativity in C, closure and maximum contrast in C were applied to the algorithm. The maximal number of components was estimated from the number of significant components (estimated from a plot of the predictive residual sum of squares (PRESS)) of a PCA model built on each pre-processed augmented data matrix using contiguous block with 8 data splits as cross-validation. The level of score value, computed as values between 0 and 1, is used as a semi-quantitative estimate of the concentration of the separated components in the mixture.

#### 2.2.3. Thermogravimetric Analysis

Thermogravimetric analysis (TGA) was performed using Discovery TGA (TA Instruments, New Castle, DE, USA). Powder samples containing physical mixtures of 15 wt% drug and 85 wt% PEO were analyzed in flame-cleansed open platinum pans by heating at 20 °C/min from 30 to 70 °C followed by an isothermal step at 70 °C for 10 min.

#### 2.2.4. Differential Scanning Calorimetry

Differential scanning calorimetry (DSC) was performed using Discovery DSC (TA Instruments, New Castle, DE, USA). Powder samples of approximately 5 mg were analyzed in closed aluminum pans by heating at 10 °C/min from 25 °C to 100 °C or 225 °C. Measurements were performed at a constant nitrogen flow rate of 50 mL/min.

#### 2.2.5. SAOS Rheometry

Small Amplitude Oscillatory Shear (SAOS) tests were performed on an AR-G2 strain-controlled rheometer fitted with an Environmental Testing Chamber (both TA Instruments, New Castle, DE, USA) using a stainless steel 25 mm plate-plate geometry, with a measurement gap of 0.8 mm.

#### 2.2.6. X-Ray Powder Diffractometry (XRPD)

X-ray powder diffraction measurements were performed using an X’Pert PANalytical PRO X-ray diffractometer (PANalytical, Almelo, the Netherlands). The following settings were used: CuKα radiation = 1.54187 Å; acceleration voltage = 45 kV; current = 40 mA; reflectance mode = 5°–35° 2θ; scan rate = 0.067° 2θ/s; step size = 0.001°.

## 3. Results and Discussion

### 3.1. Raman Spectral Analysis

**Figure 1:**
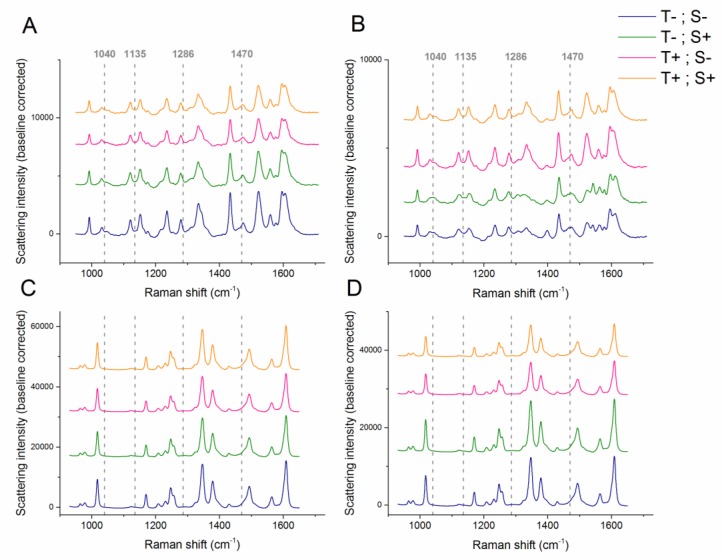
Raman spectra of (**A**) piroxicam anhydrate-polyethylene oxide (PRXAH-PEO), (**B**) piroxicam monohydrate-polyethylene oxide (PRXMH-PEO), (**C**) nitrofurantoin anhydrate-polyethylene oxide (NFAH-PEO) and (**D**) nitrofurantoin monohydrate-polyethylene oxide (NFMH-PEO) melts after 10 min of recirculation in the extruder. T−: 70 °C, T+: 90 °C, S−: 25 rpm and S+: 50 rpm. Dashed lines indicate the significant Raman shifts related to PEO.

The molten PEO displays some Raman features, with four distinct bands in the investigated range (Figure S2). However, these features are generally not visually detectable in the Raman spectra of the NF-PEO mixtures (Figure 1), except for a minor shoulder at 1470 cm^−1^, which can be detected in the spectra of the NF-PEO melt but are not present in the spectra of NFMH and NFAH (Figure S3). It should be noted that NF is a highly Raman active compound and dominates in the spectrum of a mixture with PEO.

As expected for the NF anhydrate form β, all samples of NFAH-PEO display features at 1250 cm^−1^. The positions of all major shifts are in agreement with the spectra of NFAH form β published by Aaltonen et al. [25]. Likewise, for samples of NFMH-PEO, the spectra collected at the end of the experiment, appear to show the features related to that of NFAH form β (Figure 1, Figure S3) as evident from the presence of peaks at 1018, 1250, 1349 and 1609 cm^−1^ [25]. As PRXAH form I displays Raman shifts at the same positions as the ones observed for PEO (Figure S2), it is not possible to visually detect the presence of PEO in the PRX-PEO melts. There are only minor changes in the Raman spectra of the PRXAH samples after 10 min of recirculation in the extruder, except that the Raman band peak shifts of the samples processed at 70 °C with 25 rpm appear a little sharper than those of the others. In the case of PRXMH-PEO, the temperature appears to have an influence on the Raman spectra. The samples processed at 90 °C display shifts resembling those of PRXAH form I [26], whereas the samples processed at 70 °C display a peak splitting at 1540 cm^−1^ and two-three weak, broad shifts at 1300–1350 cm^−1^, features which have previously been observed in Raman spectra of electrosprayed PRX [27]. This indicates a complex transformation pathway which justifies the use of MCR, as multiple concurrent components may be separated from this type of data analysis.

### 3.2. Multivariate Curve Resolution Models for the Polymer-Drug Systems

**Figure 2:**
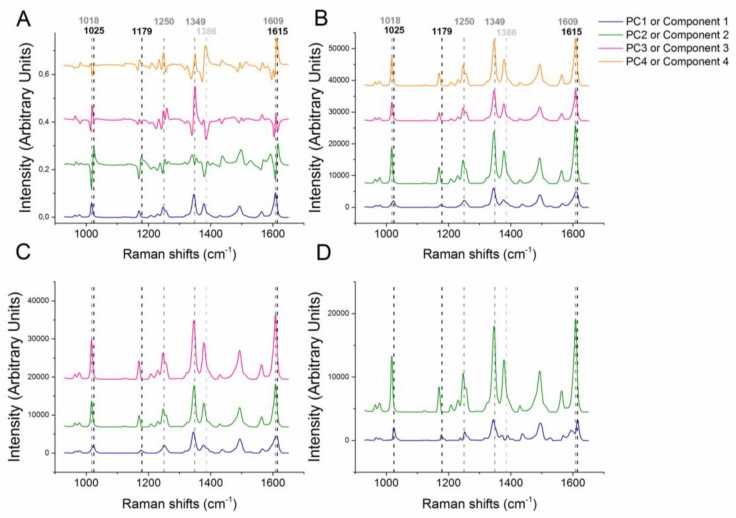
Loadings of (**A**) the Principal Component Analysis (PCA) model, (**B**) 4 component Multivariate Curve Resolution (MCR) model, (**C**) 3 component MCR model and (**D**) 2 component MCR model of the nitrofurantoin (NF) dataset. Dashed lines indicate significant shifts of NFMH (black), NFAH from β (dark grey) and NFAH form α (light grey).

The PCA modelling suggests that there are four principal components (PCs) in the NF-PEO dataset (Predictive residual sum of squares (PRESS) plot, Figure S4). The loadings on the first PC of the PCA model resemble the shifts of NFAH form β, with spectral features at 1018, 1250, 1349 and 1609 cm^−1^ (PCA model components, Figure 2A). The second PC displays three major shifted positions when compared to the first at 1025, 1179 and 1615 cm^−1^, which are characteristic shifts of NFMH form II [23]. The loadings of the third PC appear to be a mixture of PC1 and PC2, whereas the fourth has one major shift not present in the other PCs, at 1386 cm^−1^. This Raman shift value is characteristic for the NFAH form α [25], suggesting that the dehydration pathway is complex involving several kinetically trapped solid forms. The 4-component MCR model results in loadings (Figure 2B) of one component (component 1) resembling the NFMH form II well and three components with identical shift positions, but different intensity (all matching NFAH form β). The loadings of the 3-component MCR model (Figure 2C) have the second and third component loadings resembling NFAH form β and again, the first component resembling NFMH form II. In the 2-component model (Figure 2D), loadings of component 1 resemble NFMH form II and component 2 resembles NFAH form β. Thus the 2-component MCR model leads to the best estimate of the Raman spectra of the pure analytes (NF solid forms) in the NF-PEO mixtures.

**Figure 3:**
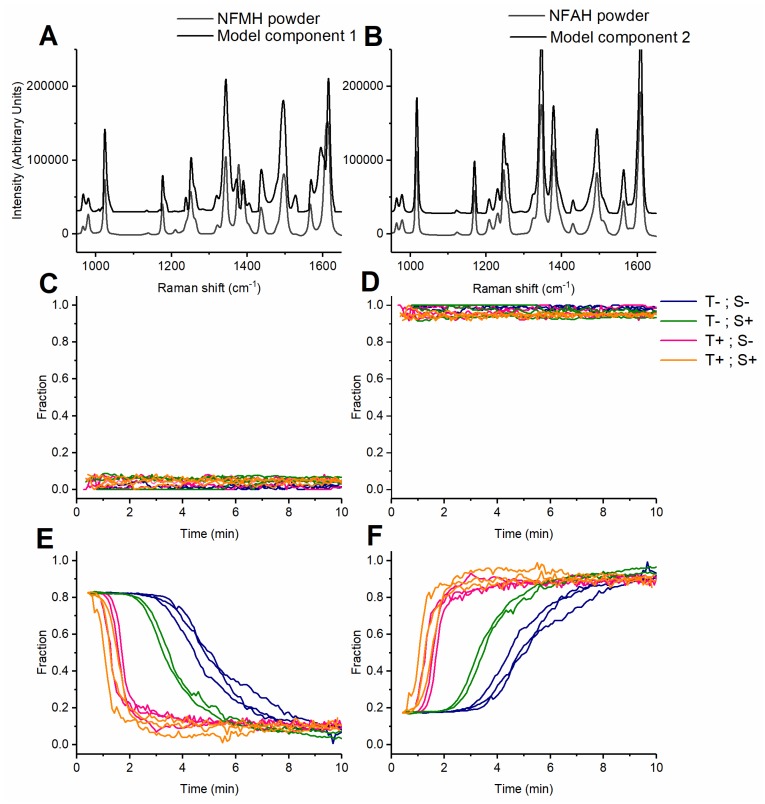
Loadings and score values (relative fraction) of a 2-component MCR model of the NF dataset collected during extrusion at different process conditions, (**A**) and (**B**) Loadings of component 1 and 2 (black) against NFMH powder and NFAH powder (gray), respectively; (**C**) score values of NFAH-PEO samples on component 1, (**D**) score values of NFAH-PEO samples on component 2, (**E**) score values of NFMH-PEO samples on component 1 and (**F**) score values of NFMH-PEO samples on component 2. Process conditions indicated with colors T−: 70 °C, T+: 90 °C, S−: 25 rpm and S+: 50 rpm.

The score values of the components in the MCR models represent the relative amount (from 0 to 1) of each component in each spectrum (which in this context each represents a specific time-point in the process). The score values of the samples containing NFAH remain stable (anhydrate) throughout the process, according to the developed 2-component MCR model (Figure 3), while the samples containing NFMH display a gradual transformation from a dominant presence of the MCR component 1 to a dominant presence of the MCR component 2. It is noticed that neither samples containing NFAH nor NFMH obtain the absolute score values 0 and 1 at any time during the process (Figure 3C–F). The results of PCA modeling of the PRX dataset suggest that these data contain variation from three principal components (PRESS plot, Figure S5).

**Figure 4:**
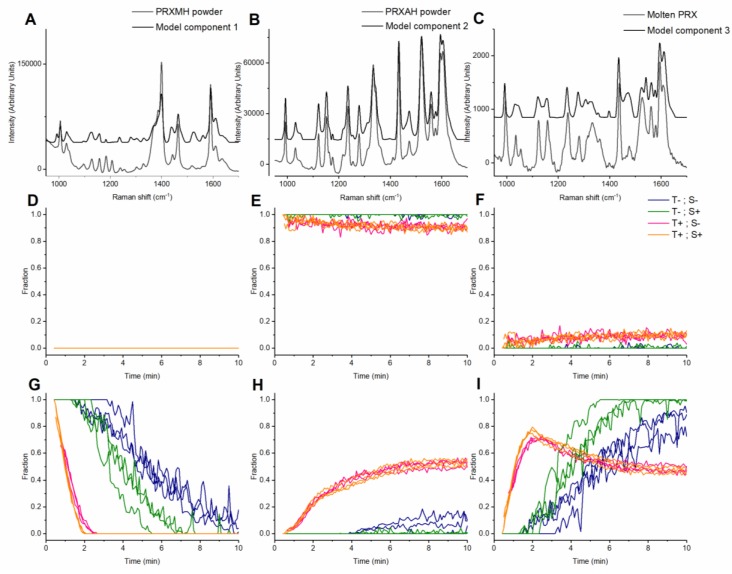
Loadings and score values (relative fraction) of a 3-component MCR model of the piroxicam (PRX) dataset collected during extrusion at different process conditions, (**A**), (**B**) and (**C**) Loadings of component 1,2 and 3 (black) against PRXMH powder, PRXAH powder and molten PRX (gray), respectively; (**D**) score values of PRXAH-PEO samples on component 1, (**E**) score values of PRXAH-PEO samples on component 2. (**F**) score values of PRXAH-PEO samples on component 3, (**G**) score values of PRXMH-PEO samples on component 1, (**H**) score values of PRXMH-PEO samples on component 2 and (**I**) score values of PRXMH-PEO samples on component 3. Process conditions indicated with colors T–: 70 °C, T+: 90 °C, S–: 25 rpm and S+: 50 rpm.

The loadings of the 3-component MCR model of the PRX dataset suggest that component 1 represents PRXMH, component 2 PRXAH form I and component 3 an unidentified solid form (Figure 4A–C) with shifts similar to those observed in the last spectra of the PRXMH samples processed at 70 °C and previously observed for electro-sprayed PRX [27]. In this model, the separation is better than for that of the NF dataset; all PRXAH samples have a score value 0 on component 1 (resembling PRXMH content) throughout the experiment (Figure 4D). This can be explained by the larger difference in the Raman spectrum of the PRX anhydrate and monohydrate species.

### 3.3. Solid Forms Detected in the Molten Polymer Mixtures

As described above, the loadings of the components 1 and 2 of the 2-component model of the NF dataset match with the Raman spectra of NFMH form II and NFAH form β, respectively (Figure 3A,B). While PCA suggests the presence of a shift at 1386 cm^−1^ which is not attributable to NFMH form I nor NFAH form β, but rather that of NFAH form α (Figure S3), this shift is not present in the resolved components in the MCR model, suggesting that it is not present in any significant amount (Figure 3A,B). In the PRX MCR models (Figure 4), component 1 resembles PRXMH, whereas component 2 matches the Raman spectra of PRXAH form I [26]. Component 3 of the MCR model on the PRX dataset is most probably related to a mixture of an amorphous PRX and (an) intermediate crystalline form(s). This conclusion is based on the general shape of the spectral features (weak and broad features), where the positions of these features seem to be a combination of the known anhydrate forms I, II and III [26]. As a consequence of random short- to mid-range order in the non-crystalline state, a Raman spectrum of an amorphous drug will consist of weak and broadened shifts at the same positions as the crystalline forms of the drug [28]. The combination of shifts is especially evident in the region of 1500–1650 cm^−1^, where 6 peaks are visible for the form resolved in component 3. The position of the shift peaks of the split signal at 1515–1545 cm^−1^ correspond to the shift position of the Raman shift at 1520 cm^−1^ present in PRXAH form III and the positions of the Raman shifts at app. 1540 cm^−1^ for PRXAH form II (Figure 4C). Likewise, the shifts in the region of 1300–1350 cm^−1^ carry characteristics of both forms III and II. Further elucidation of these phenomena could potentially be performed with faster off-line methods, such as synchrotron-based diffraction studies [24].

### 3.4. Dehydration Kinetics Derived from the MCR Model

NFMH has been reported to dehydrate through gradual dissolution into the polymer, immediately followed by precipitation of the supersaturated NF into NFAH crystals [18]. However, there was no indication from the initial model development (Figure 2) that a component representing amorphous NF could be separated in an MCR model of the NF dataset. It is visible, however, from the imperfect separation into components 1 and 2 in Figure 3C that while the NFAH would have been expected to reach a score value of 0 on the component representing NFMH, there is still some interference from an unidentified minor component in the model. Comparing the score values of component 1 and 2, the unknown solid form is combined with component 1, as NFAH is suggested to display a minor, but constant, amount of NFMH (Figure 3C). As this imperfection is affecting samples processed at 70 and 90 °C in a similar manner, it is unlikely that these differences stem from temperature-dependent artifacts in the Raman shifts of the crystalline materials; such artifacts were also not observed during inspection of the raw data. Despite having a low miscibility with PEO, a minor amorphous fraction is likely to exist in the NF melts if the transformation is a solution-mediated one. It is therefore suggested that a minor amount of amorphous NF is the unresolved component leading to an imperfect separation of NFAH and NFMH in the proposed MCR model.

From Figure 4, we gather that PRXMH dehydrates during melt processing with PEO well below the temperature reported for the dehydration of PRXMH alone at non-isothermal conditions (90–100 °C) [29,30]. Full dehydration of PRXMH at 70 °C occurs in these melt systems within 6–9 min (Table 1, whereas the same has been reported to require approximately 1000 min for PRXMH alone at 70 °C under isothermal conditions [30]. Studies of the dehydration of PRXMH alone have reported that PRXMH undergoes immediate restructuring into PRXAH upon removal of water during dehydration; that is, PRXMH is directly transformed to PRXAH form I [30,31]. However, it is observed from the MCR models and the raw Raman spectra that there is a substantial amorphous fraction of PRX present in the melt (Figure 4). Thus, in this study, dehydration appears to lead to the appearance of some amorphous PRX, likely facilitated by a relatively higher local solubility of PRXMH than PRXAH in the polymer, though dehydration through disruption of the crystal lattice as a consequence of the applied mechanical stress cannot be ruled out. Direct dehydration of PRXMH to PRXAH would be expected to occur much faster and within a relevant time-frame at 90 °C. This might explain the early formation of PRXAH, which appears to be concurrent with the formation of amorphous PRX component in the PRXMH-PEO samples processed at 90 °C. Additionally, the impact of screw speed on the recrystallization of PRXAH at 70 °C is noted; the data suggests that formation of PRXAH-I is hindered at the higher screw speed. Thus, the leveling of the PRXAH-I and amorphous PRX score values in the second half of the process for the PRXMH-PEO samples processed at 90 °C may be explained as hindering of crystallization of the amorphous PRX; the PRXAH contained in the sample at the end of processing is the product of a direct transformation of PRXMH to PRXAH form I as well as the portion crystallized from amorphous PRX (Figure 4 panel I). This opens an interesting option to use this for the control of nucleation and crystal growth rate during extrusion processes.

From the developed model, it can be observed that the PRXAH-PEO samples processed at 70 °C, PRXAH form I sustains during the duration of the experiments, whereas a minor amorphous fraction is identified at the start of the experiments at 90 °C and is constant at approximately 10–15% level during the remaining experimental time (Figure 4E,F). Maximal plasticization of the polymer is often used as an indication of the solubility limit of the drug in the polymer [32]. From frequency sweeps at 70 °C (supplementary information, Figure S6A) it can be seen that the solubility limit of PRXAH in the polymer is below 10 wt% and, considering that the plasticization of the polymer depends on the proportions of dissolved and solid drug present in the mixture [33], it may also be below 5 wt%. The lack of melting point depression of PEO in the PRX-PEO mixture observed from DSC measurements suggests that the plasticization of PEO by PRX is minimal at the studied drug concentration (Figure S6B), further emphasizing the low thermodynamic miscibility of the system [34].

Due to concurrent transformation to PRXAH, it is not possible to estimate the plasticization effect of PRXMH on PEO from frequency sweeps. However, from the Small Amplitude Oscillatory Shear (SAOS) time sweeps, it is visible that both moduli of the PRXMH-PEO melt (with 15% PRXMH) are larger than those of PEO at the beginning of the experiment and continue to increase within the timeframe where the dehydration occurs (Figure S7), which may be an effect of water release and escape from the sample, recrystallization of the anhydrate, or both. However, this change is not reflected in the force exerted on the screws during extrusion (Figure S7), where this force stays lower in the MH samples than in the extrusion of the corresponding AH samples throughout the extrusion process (Table S1). The latter suggests that for both drugs, the well-controlled amount of water released during the dehydration has a measurable and lasting plasticization effect throughout the extrusion, whereas the effects of the recrystallization process itself are negligible. This observation can also be important when the extrusion process involves mechanochemistry, such as production of co-crystal [6,35] or co-amorphous [7,8] material. Additionally, HME processing with “acidic” polymers has been reported to affect acid-base interactions with a given basic compound [36]. The presence of water can potentially affect the kinetics of all these complex transformations and the use of a hydrate compound in this context would be an elegant approach for controlled water addition to the system. In this case, the observed rate of conversion was both influenced by rotation speed and temperature (Table 1). The increase in temperature has the largest influence for both NFMH and PRXMH. The effect of each factor is constant at each level for NF, whereas there is a synergistic effect of temperature and rotation speed in the case of PRXMH. With the limited mixing capabilities of the lab scale extruder equipped with simple conical screw set without mixing elements in mind, the relative effect of individual process parameters and their interplay can well be expected to be different in processes at larger scales with more sophisticated screw designs. These scale differences further underline the importance of the application of in-line spectroscopy combined with data analysis methods that can accurately resolve multiple species in complex matrix formulations, such as MCR-ALS, during both formulation and process development.

From Table 1 it is also observed that the time needed for a complete water removal recorded from TGA of the material do not match with that of the complete dehydration recorded by Raman spectroscopy; measured as the complete disappearance of PRXMH (component 1 of the model presented in Figure 4). This faster dehydration observed in the TGA measurements is likely a consequence of the lack of continuous mechanical stress during the TGA measurement, possibly leading to incomplete dehydration from less intimate mixing of the drug and the polymer. Further insight to the dehydration mechanism and kinetics may be achieved from the combination of multiple *in-line* measurements techniques, as has been demonstrated earlier in the visualization of a pharmaceutical wet granulation process [37]. As the release of small amounts of water apparently plasticize the melt, in-line measurements of water content and the force/torque together with Raman spectroscopy would provide a useful combination for process monitoring and control purposes.

### 3.5. Critical Evaluation of the Dehydration Mechanism in Molten Polymer Systems

As shown in Figure 2, it was not possible to create a 3-component MCR model of the NF-PEO dataset that could separate the spectral data in a meaningful way. However, as discussed above, it appears from the 2-component MCR model that there is an unresolved third NF solid form present. The lack of separation may be due to the Raman shifts of the different NF solid forms being too close for accurate modeling of the amorphous form. It may also be that due to the intense scatter of the crystalline NF, the shifts of the coexisting amorphous NF are suppressed beyond the detection limit of the model, similar to how the PEO shifts are overshadowed by the intensity of the shifts from the drugs. Including samples with higher amorphous content of NF might make it possible to separate a component representing the amorphous fraction. It should be further noted that we cannot exclude the possibility of presence of minute amounts of kinetically trapped other polymorphic forms, in this case the presence of NFAH form α. Similarly, Scaramuzza et al. has reported the complex dehydration mechanism of carbamazepine dihydrate (CBZ DH) during polymer-assisted grinding (POLAG), where the dehydrated CBZ DH followed Ostwald’s rule of stages resulting in a mixture of higher energy anhydrate and the stable CBZ anhydrate forms [38].

The produced MCR models are not able to take into account the solid form of the material within the first 40–60 s of the process due to the position of the probe at the bottom of the barrel leading to a lag time in the time before the melt is properly distributed in the barrel. Another limitation in the sampling is the inherent heterogeneity of the melt, it being a multiphase system, which makes representative sampling difficult [39]. The effect of this system property is seen in the fluctuation of the score values of the NF and PRX models, a fluctuation which is resulting from sampling different parts of the melt stream, where the distribution of solid forms is different from that of the previous sample. Consequently, larger fluctuations are seen for the slow conversion of PRXMH at 70 °C than is seen for the fast dehydration of PRXMH at 90 °C, where the rapid transformation would be expected to lead to higher sample homogeneity. In this respect, the large number of sampling time-points provides in-line measurements with another advantage over sampling for off-line analysis; that is, the possibility to detect and verify inhomogeneity in the melt during processing. For such applications, NIR spectroscopy, which allows for improved spatial resolution over Raman spectroscopy, has been demonstrated to be a useful tool [40].

## 4. Conclusions

This study confirms that dehydration during hot melt extrusion may occur at lower temperatures than expected based on the laboratory-based off-line thermal analysis. This observation illustrates the complex interplay between thermal and mechanical stress along with excipient interactions affecting the solid form diversity of the drug after the melt extrusion process. The increased screw speed was found to increase the presence of high energy solid forms of PRX and similar behavior was observed during extrusion of NF with PEO. It was also observed that dehydration during extrusion provides a method for well-controlled addition of water during processing, which resulted in a measurable plasticization effect. Process analytics will be critical element and enabling tool in future manufacturing of innovative pharmaceutical products based on extrusion processes.

## Figures and Tables

**Table 1:** End-point of transformation (MH- > AH) in minutes (defined as 95% change in MH containing samples) as calculated from the MCR models of in-line Raman spectroscopy and from TGA experiments, reported as average values with standard deviation in brackets, *n* = 3.

Model System		NF		PRX	
Process Temperature (°C)		70	90	70	90
Melt extrusion experiments (MCR models)	Screw speed (rpm)	End-point of transformation (MH to AH) (min) (std. dev.)			
	25	8.4 (0.4)	3.4 (0.5)	8.6 (0.5)	2.4 (0.0)
	50	7.1 (-) *	2.3 (0.7)	6.0 (0.7)	1.8 (0.0)
TGA experiments		4.2 (0.1)	-	4.8 (0.9)	-

* One sample missing, average of two values.

## Supplementary Materials

The following are available online at https://www.mdpi.com/1999-4923/12/2/116/s1, Figure S1: XRPD patterns of raw materials and their CSD references, Figures S2 and S3: Raman spectra of raw materials; Figures S4 and S5: Predictive residual sum of squares of PCA models, Figure S6: Frequency sweeps and differential scanning calorimetry results, Figure S7: Rheometry and differential scanning calorimetry results of drug-polymer mixtures and force measurement results of extrusion of the drug-polymer mixtures (PDF); Table S1. Force exerted on screws in Newtons at timepoint 600 s during extrusion, reported as average values with standard deviation in parentheses, *n* = 3.
